# CHITRA: an interactive visualization tool for comparative genomic rearrangement analysis

**DOI:** 10.1093/bioadv/vbag200

**Published:** 2026-07-17

**Authors:** Pranjal Pruthi, Ajay Bhatia, Preeti Agarwal, Nityendra Shukla, Lata Rani, Jitendra Narayan

**Affiliations:** CSIR-Institute of Genomics and Integrative Biology, New Delhi 110007, Delhi, India; CSIR-Institute of Genomics and Integrative Biology, New Delhi 110007, Delhi, India; Academy of Scientific and Innovative Research (AcSIR), Ghaziabad 201002, Uttar Pradesh, India; CSIR-Institute of Genomics and Integrative Biology, New Delhi 110007, Delhi, India; Academy of Scientific and Innovative Research (AcSIR), Ghaziabad 201002, Uttar Pradesh, India; CSIR-Institute of Genomics and Integrative Biology, New Delhi 110007, Delhi, India; Genomics Facility Centralized Core Research Facility (CCRF), AIIMS, New Delhi 110029, Delhi, India; CSIR-Institute of Genomics and Integrative Biology, New Delhi 110007, Delhi, India; Academy of Scientific and Innovative Research (AcSIR), Ghaziabad 201002, Uttar Pradesh, India

## Abstract

**Motivation:**

The increasing availability of chromosome-scale genome assemblies has fuelled a renewed interest in studying chromosomal evolution and rearrangements. Synteny visualization plays a critical role in understanding genome organization, structural variations, and evolutionary relationships. However, existing tools often have steep learning curves, produce static plots, or are limited in their ability to analyse multiple genomes simultaneously. There is a growing need for an intuitive and interactive visualization tool that can effectively explore syntenic relationships and chromosomal rearrangements.

**Results:**

Here, we present CHITRA, a web-based interactive tool designed to visualize synteny blocks, chromosomal rearrangements, and breakpoints in both linear and circular styles. CHITRA-enables real-time exploration of genome structural variations with an intuitive graphical interface, customizable visualization options, and high-resolution export capabilities for publication-ready figures. The tool supports chromosome- and scaffold-level assemblies and allows users to filter, highlight, and interactively examine syntenic relationships.

**Availability and implementation:**

CHITRA is freely available at https://chitra.bioinformaticsonline.com/, with comprehensive documentation at https://chitra.bioinformaticsonline.com/docs. The source code is open-source and accessible on GitHub at https://github.com/pranjalpruthi/CHITRA.

## 1 Introduction

Chromosomal rearrangement plays a crucial role in genome evolution, understanding evolutionary histories, and adaptation mechanisms of organisms of specific species or lineages (Crombach and Hogeweg 2007, Kirkpatrick 2010). In recent years, chromosomal evolution studies have seen a resurgence in interest owing to advances in sequencing technologies, facilitating the release of high-quality chromosome-scale whole-genome assemblies by large sequencing projects (Rhie *et al.* 2021), thus unlocking the potential for studying chromosome rearrangement events.

Synteny plays an important role in genome organization, and the visualization of synteny blocks is important for identifying structural variations, evolutionary relationships, and gene conservation across taxa (Ghiurcuta and Moret 2014). Synteny blocks can be visualized in various ways, with circular visualization such as Circos plots (Krzywinski *et al.* 2009) being popular. However, Circos plots have notable limitations, in being difficult to interpret small synteny blocks and chromosomal rearrangements as well as comparative analysis being limited to two genomes, thus leading to an increased usage of linear plots. Linear plots allow for identification and visualization of microsynteny blocks and small structural variations, such as inversions or translocations. More importantly, linear plots allow multiple genomes to be visualized. Various tools exist for linear genome visualization, such as GENESPACE (Lovell *et al.* 2022), GenomicRanges (Lawrence *et al.* 2013), karyoploteR (Gel and Serra 2017), and plotgardener (Kramer *et al.* 2022). Recently, a few synteny visualization packages have been developed and have gained popularity, such as syntenyPlotteR (Quigley *et al.* 2023), ShinySyn (Xiao and Lam 2022), and NGenomeSyn (He *et al.* 2023). However, as with the previous software, each of these packages has their own features and limitations (Table 1), with a common one being that all of them produce static plots, thus lacking the functionality that graphically interactive linear visualizations can provide.

**Table 1:** Comparative overview of various features available across various synteny visualization tools.

Feature	CHITRA	Circos	SynVisio	SimpleSynteny	Synima	syntenyPlotteR	Mizbee	gggenomes	GENESPCE
**Web interface**	Yes	No	Yes	Yes	No	No	No	No	No
**Visualization type**	Linear, circular	Circular	Linear, dot plot	Linear	Linear	Linear	Linear, circular	Linear	Linear, dot plot
**Collaborative sharing**	Yes	No	No	No	No	No	No	No	No
**Structural variation annotation**	Yes	No	No	No	No	No	Partial	No	Partial
**Chromosome filter**	Yes	No	Yes	No	No	No	Yes	No	Partial
**Genome filter**	Yes	No	Yes	No	No	No	No	No	No
**Strand filter**	Yes	No	Yes	No	No	No	No	No	No
**Chord/block filter**	Yes	No	Yes	No	No	No	Partial	No	No
**Breakpoint visualization**	Yes	No	No	No	No	No	No	No	No
**Multi-scale exploration**	Yes	No	Yes	No	No	No	Yes	Yes	Yes
**Mutation tags**	Yes	No	No	No	No	No	No	No	Partial
**Export**	Yes (PNG/SVG)	Yes (PNG/SVG)	Yes (PNG/SVG)	Yes (PNG/PDF)	Yes (PDF)	Yes (PNG/PDF)	Partial	Yes (PNG/PDF)	Yes (PNG/PDF)

To overcome this problem, we created the Chromosome Interactive Tool for Rearrangement Analysis (CHITRA) to provide detailed and interactive genomic data visualization, allowing for visualization of synteny blocks, chromosomal rearrangements and breakpoints in both linear and circular visualization styles. CHITRA provides an intuitive and user-friendly graphical user interface (GUI), along with the ability to produce publication-ready plots with a plethora of interactive visualization options, featuring real-time hover and click interactions for synteny block exploration, dynamic multi-axis filtering at various levels of genome organization, direct feature table conversion, and can be used with both chromosome-scale and scaffold-scale genome assemblies. CHITRA is released under the MIT licence, and is available for use at https://chitra.bioinformaticsonline.com/, with comprehensive documentation hosted at https://chitra.bioinformaticsonline.com/docs. The source code can be accessed on GitHub at https://github.com/pranjalpruthi/CHITRA.

## 2 Implementation

The CHITRA architecture is developed using Next.js (https://github.com/vercel/next.js), a React-based framework supporting server-side rendering and static site generation to handle complex genomic data efficiently. The interface employs ShadCN UI (https://github.com/shadcn-ui/ui), Tailwind CSS (https://github.com/tailwindlabs/tailwindcss), and Framer Motion (https://motion.dev/).

Data visualization relies on D3.js (https://github.com/d3/d3/), rendering synteny plots, chord diagrams, and ribbon-based representations of genomic conservation. High-resolution export options are provided via jsSVG (https://github.com/coldpour/jssvg), enabling publication-ready outputs in vector (SVG) or raster formats (PNG, JPG). Google Gemini 2.5 Pro (Google DeepMind) was used as a coding assistant to aid in the integration and adaptation of UI components from the ShadCN/UI library into the CHITRA web application (e.g. interactive data tables, visualization panels, and navigation controls). AI was not used for scientific analysis, data interpretation, algorithm design, or manuscript writing. All AI-assisted code was reviewed, tested, and validated by the authors, who take full responsibility for the integrity and accuracy of the work.

## 3 Features

**Figure 1:**
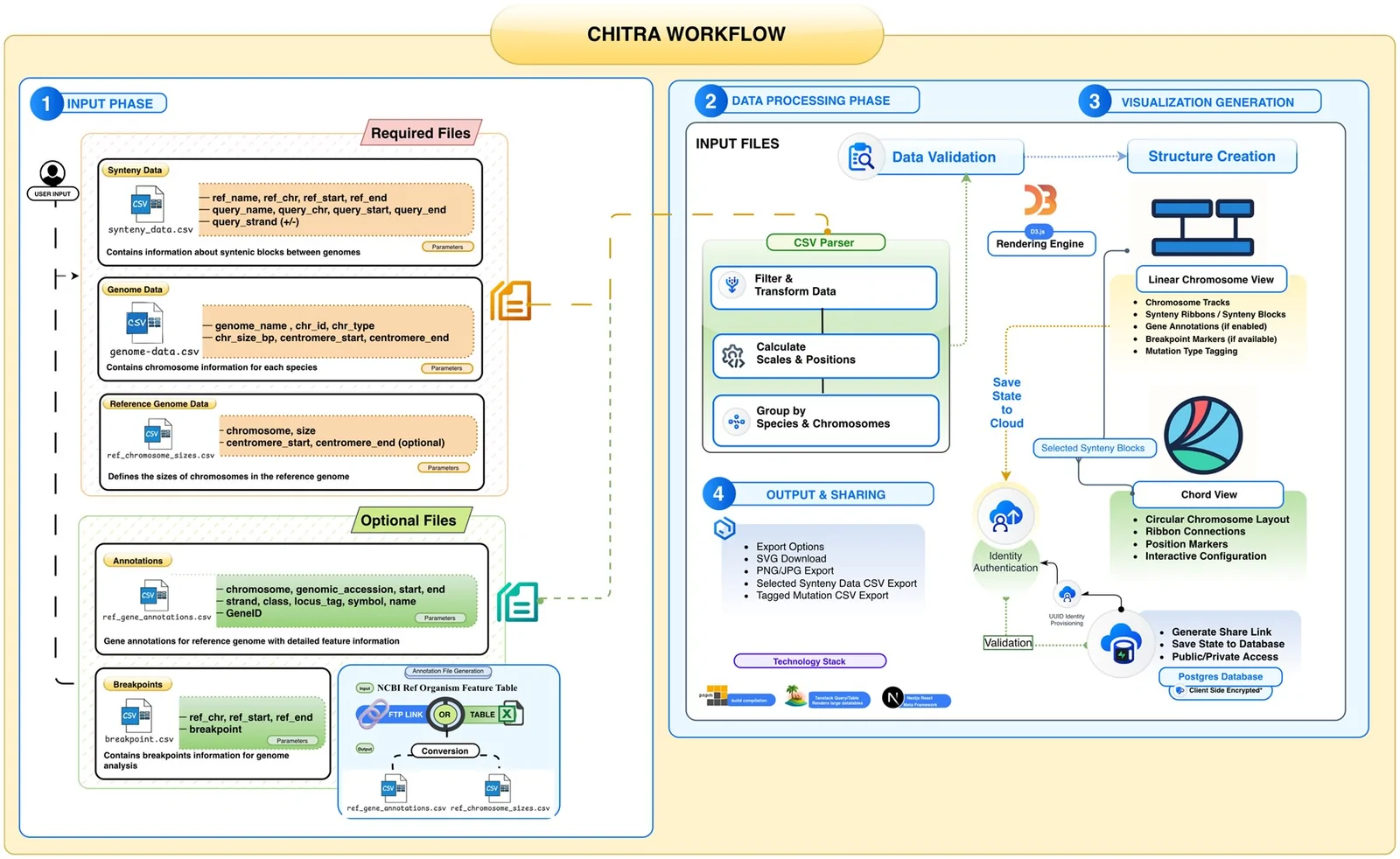
Workflow of CHITRA showcasing input data processing, feature table conversion, and an interactive visualization of synteny blocks, chromosomal breakpoints and data export options.

CHITRA requires three input files (Fig. 1), all in CSV format: (i) synteny data containing pairwise synteny blocks between genomes, (ii) genomic data for genome information, and (iii) reference chromosome size data. The synteny data can be generated by performing genome alignment and identifying synteny blocks by using tools like SyntenyTracker (Donthu *et al.* 2009). Additionally, two optional input files can also be added to enhance the visualization: gene annotations and breakpoint data. The user can use a variety of gene annotation software to perform annotations and identify breakpoints. CHITRA can also process NCBI feature data table files to generate the required annotations.

**Figure 2:**
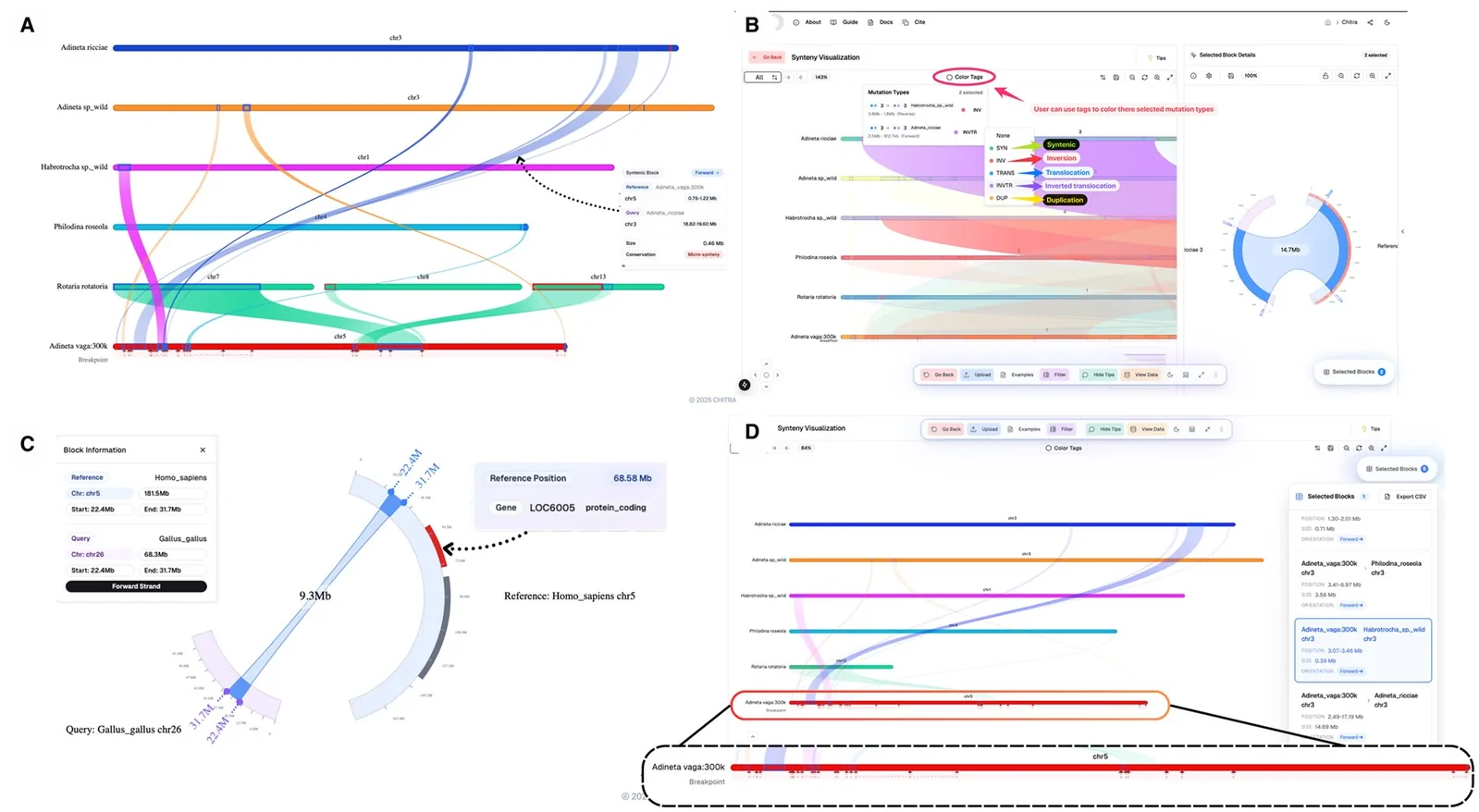
An overview of various visualization styles in CHITRA. (A) Linear synteny visualization plot displaying various chromosomal rearrangements, depicted through a Sankey plot. (B) Optional colour-tag feature that allows users to customize the ribbon colours to denote distinct mutation types (e.g. inversions, translocations, duplications, etc.). (C) Detailed chord map displaying synteny blocks between a query and reference genome. (D) Linear synteny plot enhanced with breakpoint mapping, with the red arrows indicating chromosomal breakpoints across chr5 of *Adineta vaga*.

Users can either download these feature tables from the NCBI RefSeq FTP server or provide a URL for automated processing. Since CHITRA is a fully client-side web application, its performance ceiling scales natively with the user’s local hardware configuration, however, we have optimized performance for larger eukaryotic genome visualization through SVG virtualization, viewport culling and aggressive background data filtration by rendering only specific subsets of regions the user is focused on, ensuring minimum memory load and allowing CHITRA to be used on machines with lower hardware configurations. Finally, the user can utilize both the documentation and example datasets to tailor their data to that required by CHITRA. The data can be visualized in two main ways: (i) a linear synthetic view and (ii) a circular chord map. Using example datasets (Simion *et al.* 2021), we demonstrate chromosomal rearrangements between multiple genomes (Fig. 2).

## 4 Results

CHITRA can be used beyond visualization as a practical tool for downstream comparative and evolutionary analyses. It supports (i) evolutionary studies and comparative genomics by enabling interactive exploration of conserved synteny blocks across taxa, which can help infer genome conservation patterns and broad-scale rearrangement histories and (ii) structural variation analysis by helping users visually identify and interpret large rearrangements such as inversions, translocations, and duplications through synteny block orientation and breakpoint-supported patterns.

### 4.1 Interactive synteny visualization

We use a linear visualization interface to visualize inter- and intra-chromosomal rearrangement events. Synteny blocks between genomes are represented as connected interactive ribbons (Fig. 2A). Hovering the cursor over a synteny block reveals the size category of the block, calculated as:


$$(\frac{\mathrm{current}\;\mathrm{block}\;\mathrm{size}}{\mathrm{largest}\;\mathrm{block}\;\mathrm{size}})\times 100.$$

The categories are based on size and defined as follows: large blocks are those >10 Mb, medium regions are >5 Mb and up to 10 Mb, small regions are >1 Mb and up to 5 Mb, and microregions are 1 Mb or less. The progress bar below the category information in the tooltip provides a visual representation of the hovered synteny block’s size relative to the largest synteny block in the current dataset. Users can customize the ribbon colours to represent the different types of rearrangement mechanisms, as demonstrated in Fig. 2B. Additionally, users can select specific reference chromosomes, choose specific genomes to include in the visualization, filter connected query chromosomes, and select individual chromosomes from each genome. CHITRA also supports scaffold or contig-level assemblies. Finally, users can also filter the visualization to display forward strand synteny blocks, reverse strand synteny blocks, or both, which is enabled by default, providing flexibility in analysing genomic orientations.

### 4.2 Chord map visualization

The chord map offers a detailed circular visualization of synteny blocks between genomes (Fig. 2C). When users select a specific synteny block, the chord map displays comprehensive information, including the reference chromosome, the corresponding chromosomes in query genomes, their block sizes, locations, and orientations. This visualization style is particularly effective for analysing complex genomic relationships and identifying structural variations between genomes. Additionally, the chord map includes interactive features allowing users to filter, highlight, and examine specific syntenic relationships in detail.

### 4.3 Annotations and breakpoint mapping

The capabilities of CHITRA can be further enhanced with the inclusion of gene annotation and chromosomal breakpoint data. Gene annotations can be visualized within the detailed chord diagram bands, enabling gene-level identification of synteny blocks (Fig. 2B). Additionally, the breakpoint mapping functionality provides a specialized visualization for chromosomal breakpoints, highlighting them by red arrows in the linear synteny plot and as red lines on the reference genome chord in the chord map. Incorporation of this additional information can provide insights into revealing gene density patterns and functional hotspots (Lemaitre *et al.* 2009), validation of syntenic relationships through conserved gene order, helping identify regions prone to rearrangements (Murphy *et al.* 2005), and understanding selective pressures shaping genome organization (Lemaitre *et al.* 2009).

## 5 Conclusions

We developed CHITRA, a web-server designed to interactively visualize syntenic relationships, chromosomal rearrangements and breakpoints in both linear and circular visualization styles. CHITRA is highly customizable and allows for various types of labelling and viewing. Additionally, CHITRA can visualize chromosome-level as well as scaffold-level assemblies, making it a versatile visualization software. CHITRA is available at https://chitra.bioinformaticsonline.com/chitra, and a comprehensive manual is available at https://chitra.bioinformaticsonline.com/docs.

## Data Availability

The source code of CHITRA is publicly available in *GitHub* at https://github.com/BioinformaticsOnLine/CHITRA. The data used for the visualization in Fig. 2 is available at PRJNA680543. No addition datasets were generated or analysed during current studies.
